# Are Patients with Active Cancer and Those with History of Cancer Carrying the Same Risks of Recurrent VTE and Bleeding While on Anticoagulants?

**DOI:** 10.3390/cancers12040917

**Published:** 2020-04-09

**Authors:** Corinne Frere, Benjamin Crichi, Manon Lejeune, Jean-Philippe Spano, Nicolas Janus

**Affiliations:** 1 Institute of Cardiometabolism and Nutrition, INSERM UMRS_1166, GRC 27 GRECO, Sorbonne Université, F-75013 Paris, France; corinne.frere@aphp.fr (C.F.); manon.lejeune@aphp.fr (M.L.); 2 Department of Haematology, Pitié-Salpêtrière Hospital, Assistance Publique Hôpitaux de Paris, F-75013 Paris, France; 3 Department of Internal Medicine, Autoimmune and Vascular Disease Unit, Saint-Louis Hospital, Assistance Publique Hôpitaux de Paris, F-75010 Paris, France; benjamin.crichi@aphp.fr; 4 INSERM Institut Pierre Louis d'Epidémiologie et de Santé Publique (iPLESP), Sorbonne Université, F-75013 Paris, France; jean-philippe.spano@aphp.fr; 5 Department of Medical Oncology, Pitié-Salpêtrière Hospital, Assistance Publique Hôpitaux de Paris, F-75013 Paris, France; 6 Global Thrombosis Strategy, Medical Affairs, Leo Pharma A/S, 2750 Ballerup, Denmark

**Keywords:** cancer-associated-thrombosis, anticoagulant, active cancer, history of cancer

## Abstract

Direct oral anticoagulants (DOAC) are now recommended for the treatment of cancer-associated thrombosis (CAT) based on the results of dedicated trials demonstrating that DOAC are non-inferior to low molecular weight heparins in preventing recurrent venous thromboembolism (VTE) in this population. The definition of “cancer patient” differs substantially among studies. Whether patients with active cancer and those with a history of cancer (HOC) carry the same risks of recurrent VTE and bleeding remains unclear. Few studies reported data on the efficacy and safety of anticoagulants according to active cancer or HOC categories. While in subgroup analyses of EINSTEIN and HOKUSAI the rates of recurrent VTE and bleeding did not differ between these categories, results from a subgroup analysis of AMPLIFY, from HOKUSAI-Cancer, and from the COMMAND cohort suggest that HOC patients might have a lower bleeding risk than active cancer patients. Whether the inclusion of HOC patients in CAT studies might introduce some bias by decreasing the rates of both recurrent VTE and bleeding remains an unanswered issue since no dedicated prospective study addressed this question. A strict definition of active cancer should be used in further trials.

## 1. Introduction

Venous thromboembolism (VTE) is a common complication and the second leading cause of death in cancer patients after malignancy progression [1]. This multifactorial disease results from a complex interplay involving inherited and acquired risk factors. Factors contributing to the VTE risk in cancer patients are usually categorized into patient-related factors, cancer-related factors, and treatment-related factors (Table 1) [1].

The risk for VTE differs widely according to the cancer type, pancreatic cancer carrying the highest risk of VTE among all malignancies [2].

**Figure 1:**
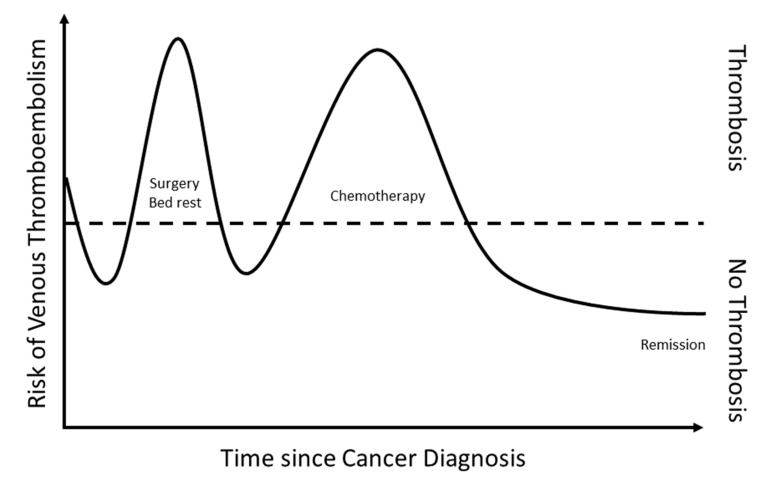
Overall risk for VTE throughout the course of cancer disease.

Importantly, the overall risk for VTE varies for a single cancer patient throughout the course of cancer disease (Figure 1). First, there is a time-dependent variation in the risk of VTE, patients having the highest risk during the first three months after cancer diagnosis [3]. Second, cancer therapies, including surgery and chemotherapy, are particularly strong risk factors for VTE occurrence. Patients receiving chemotherapy, hormonal therapy, or targeted agents are at seven-fold increased risk for VTE as compared to the non-cancer population [4]. Supportive care measures, which are commonly used in ambulatory cancer patients, such as the administration of erythroid-stimulating agents and of blood transfusions, are also associated with an increased risk of VTE [5,6].

One can assume that the risk for VTE reduces over time in patients with cancer cured. Accordingly, a recent large population-based cohort study found that the absolute incidence of VTE was highest closer to the time of cancer diagnosis and decreased over time [7]. However, in this study, cancer survivors still carried a substantially higher risk of VTE compared to the general population for at least five years after diagnosis for most cancer types, although this risk attenuated over time [7].

Management of VTE is challenging in cancer patients who are more likely to experience VTE recurrence and major bleeding events while on anticoagulants relative to non-cancer patients [8].

Long-term use of low molecular weight heparins (LMWH) has been the standard of care for the treatment of cancer-associated thrombosis (CAT) over the past fifteen years, based on the results of five randomized controlled trials (RCT) demonstrating that LMWH are more effective and at least as safe as standard therapy with LMWH followed by vitamin K antagonists (VKA) [9,10,11,12,13].

Evidence supporting the use of direct oral anticoagulants (DOAC) have recently become available with the publication of two dedicated noninferiority trials and one large pilot study [14,15,16] demonstrating that DOAC are non-inferior to LMWH for the prevention of recurrent VTE in this specific population, but confer a higher risk of bleeding in patients with gastrointestinal and genitourinary malignancies, which provided a significant advance in the field [17,18]. In light of these results, both DOAC and LMWH are now recommended as first-line option for the treatment of CAT by all recent guidelines [19,20,21].

However, there is substantial variation in the definition of “active cancer” among studies conducted since the early 2000s, and it should be noted that the most recent trials also included a significant proportion of patients with a history of cancer. 

## 2. Various Definitions of Active Cancer Have Been Used in Clinical Trial for the Treatment of CAT

A Broad definition of active cancer has been proposed by the Haemostasis and Malignancy Scientific and Standardization Committee (SSC) of the International Society on Thrombosis and Haemostasis (ISTH) [22]. The Haemostasis and Malignancy SSC defines “active cancer” as cancer diagnosed within the previous six months, recurrent, regionally advanced or metastatic cancer, cancer for which treatment had been administered within six months, or hematological cancer that is not in complete remission. 

While in the landmark CLOT [10] and CATCH [13] studies, and more recently in the pilot (Anticoagulation Therapy in Selected Cancer Patients at Risk of Recurrence of Venous Thromboembolism) SELECT-D study [15], “active cancer” was defined according to the Haemostasis and Malignancy SSC broad definition [22], the HOKUSAI VTE Cancer trial [14] and the recently published CARAVAGGIO trial [16] allowed including patients with “Cancer diagnosed within 2 years before enrolment” (i.e., with a “history of cancer”).

Interestingly, in the HOKUSAI VTE Cancer study [14], while more than 97% of patients had “active cancer” at study entry (approximatively 52% of them having metastatic disease and 71% of them receiving anticancer therapy), 233 out of 1050 (22%) randomized patients had their cancer “cured” at the time of the index VTE event. Similarly, more than 97% of patients had “active cancer” at study entry in the CARAVAGGIO trial [16]. However, the rate of cancer “cured” at the time of the index VTE event was not reported. 

Whether these two groups of cancer patients carry the same risk of recurrent VTE and bleeding and whether the inclusion of patients with a history of cancer in these studies might have introduced some bias by decreasing the rates of recurrent VTE and bleeding remain unanswered issues. 

## 3. Risk of Recurrent VTE and Bleeding in Patients with an “Active Cancer” and in Those with a “History of Cancer”

Few studies have separately reported the rates of recurrent VTE and bleeding in patients with an “active cancer” and in those with a “history of cancer”.

Most available data were derived from secondary analyses of pivotal phase III clinical RCT which have demonstrated that direct Xa inhibitors are non-inferior to standard therapy with LMWH followed by Vitamin K antagonists (VKA) for preventing recurrent VTE in the general patient population, with similar or lower rates of bleeding (EINSTEIN-PE and-DVT for rivaroxaban, AMPLIFY for apixaban, and HOKUSAI for edoxaban) [23,24,25,26]. Subgroup analyses of all trials led to the same conclusions that direct Xa inhibitors might be as effective as standard therapy with LMWH followed by warfarin for the prevention of recurrent VTE in cancer patients, with less or similar rates of bleeding [27,28,29].

Importantly, a small number of cancer patients (3–9%) were included in these RCT and definitions for “active cancer” differed substantially among the trials.

In EINSTEIN-PE and-DVT [27], the investigators reported the presence of “cancer” at study entry on the case report form. Non-melanoma skin cancers were not excluded. In the post-hoc analysis, cancer patients were further reclassified as follows: cancer at baseline (defined as a diagnosis of cancer occurring within six months before enrolment, any cancer treatment within the previous six months, or recurrent or metastatic cancer), active cancer during the study (defined as a new diagnosis of cancer or recurrence of cancer after randomization), and history of cancer (defined as any cancer not meeting the criteria of active cancer, i.e., having previously had cancer that was either cured or in remission). Overall, 463 (6%) patients had an active cancer at baseline, 193 (2%) patients had a newly diagnosed cancer during the study, and 469 (8%) patients had a history of cancer.

In the AMPLIFY subgroup analysis [28], active cancer was defined as cancer diagnosed or treated within the past six months, while history of cancer was defined as cancer diagnosed for more than six months in the absence of any cancer treatment, including surgery, radiotherapy, chemotherapy, hormonal therapy, palliative care, or combined modality therapy. Nonmelanoma skin cancer were not excluded. Among the 5395 patients included in AMPLIFY, 169 (3.1%) had an active cancer at baseline and 365 (6.8%) had a history of cancer without active cancer.

Unlike in the AMPLIFY trial, no specific definition of cancer was given in the protocol of the HOKUSAI trial [29]. In total, 771 patients (9% of the overall population) were categorized as having a cancer at enrolment by the study physician, based on his clinical judgment.

All patients initially categorized as cancer patients were further reviewed post hoc and re-classified as having an active cancer in the case of solid measurable cancer other than non-melanoma skin cancer, or hematological malignancy not in remission.

Neither in AMPLIFY nor in HOKUSAI data from patients with new diagnosis of cancer or recurrence of cancer after randomization were reported separately. 

The rates of recurrent VTE and bleeding reported in subgroups analyses of (Oral rivaroxaban for the treatment of symptomatic pulmonary embolism) EINSTEIN-PE, (Oral rivaroxaban for symptomatic venous thromboembolism) EINSTEIN-DVT, (Oral apixaban for the treatment of acute venous thromboembolism) AMPLIFY, and (Edoxaban versus warfarin for the treatment of symptomatic venous thromboembolism) HOKUSAI trials are summarized in Table 2.

The rate of recurrent VTE was generally numerically similar in patients with a history of cancer and those with an active cancer at study entry, except in the AMPLIFY study (1.1% vs. 3.7%) [28]. However, no p-value for between groups comparison was reported in the publication. Finally, in AMPLIFY, the rate of major and clinically relevant non-major bleeding (CRNMB) seemed to be numerically higher in patients with an active cancer compared to those with a history of cancer in both treatment groups. Indeed, the rates of major and CRNM bleeding were 22.5% and 12.6% in patients with an active cancer in the control and experimental arms, respectively, compared to 15.1% and 6% in those with a history of cancer. However, once again, no p-value for between groups comparison was reported [28].

Importantly, in subgroups analyses of EINSTEIN-PE, EINSTEIN-DVT, AMPLIFY, and HOKUSAI, the rates of recurrent VTE were lower in the control groups of cancer patients (4–9%) than the 15.7% and 7.2% reported in the CLOT and CATCH studies, respectively [10,13]. The most likely explanation is that patients with active cancer included in these pivotal trials may have had less aggressive or extensive disease than those entered in dedicated studies, and therefore may not truly reflect the real-life cancer patient population. Less than 30% of them had metastatic disease at study entry (compared to 67% and 55% in CLOT and CATCH, respectively) and less than 40% of them were receiving systemic anticancer therapy (compared to 77% and 52% in CLOT and CATCH, respectively) [10,13]. In all cases, patients with a life expectancy shorter than six months and those for whom long-term treatment with LMWH therapy was anticipated were excluded from the studies. Therefore, if a true difference regarding the risks of recurrent VTE or bleeding exists between patients with an active cancer and those with a history of cancer, this difference might have been underestimated.

Recently, the COMMAND VTE (COntemporary ManageMent AND outcomes in patients with Venous ThromboEmbolism) Registry assessed clinical characteristics, management strategies, and outcomes in patients with acute symptomatic VTE in real-life [30]. This multicentric registry enrolled 3027 consecutive patients with acute symptomatic VTE in Japan between 2010 and 2014. Patients were divided into three groups: patients with active cancer (defined as those receiving treatment for cancer, those scheduled to undergo surgery for cancer, and those with metastasis or terminal cancer at the time of VTE diagnosis; *n* = 695, 23%), patients with history of cancer (*n* = 243, 8%), and patients without history of cancer (*n* = 2,089, 69%). Among patients with active cancer, 49% had metastatic or terminal stage disease, and 55% were receiving chemotherapy. Warfarin was used for long-term treatment of VTE in most cases (97.4%).

The cumulative five-year incidences of recurrent VTE were reported to be significantly higher in patients with active cancer compared to those without history of cancer (17.7% vs. 8.6%, adjusted HR 2.94, 95% CI 2.20–3.92, *p* < 0.001), while no difference was observed between patients with history of cancer and those without history of cancer (10.2% vs. 8.6%, adjusted HR 1.47, 95% CI 0.87–2.36, *p* = 0.15). Similarly, the cumulative five-year incidences of major bleeding were found to be significantly higher in patients with active cancer compared to those without history of cancer (26.6% vs. 9.3%, adjusted HR 2.48, 95% CI 1.9–3.23, *p* < 0.001). On the contrary, no difference in major bleeding was observed between patients with history of cancer and those without history of cancer (8.8% vs. 9.3%, adjusted HR 1.01, 95% CI 0.60–1.59, *p* = 0.97). Although no head-to-head comparison between patients with active cancer and those with history of cancer was performed in this study, these results strongly suggest that the risks of both recurrent VTE and major bleeding might not be similar in these two subgroups of patients. Furthermore, patients with active cancer tended to be more exposed to fatal bleeding compared to those with history of cancer (6.1% vs. 1.1%; *p* = 0.1). 

In the HOKUSAI VTE Cancer study [14], the rates of recurrent VTE did not differ between patients with active cancer and patients with history of cancer: recurrent VTE occurred in 33 out of 397 (8.3%) patients with cancer not cured vs. in 8 out of 125 (6.4%) patients with cancer cured in the edoxaban arm, and in 48 out of 410 (11.7%) patients with cancer not cured vs. in 11 out of 114 (9.6%) patients with cancer cured in the LMWH arm. However, the rate of major bleeding was higher in patients with cancer not cured (29 out of 397, 7.3%) than in patients with cancer cured (3 out of 125, 2.5%) in the edoxaban arm, and tended to be higher in patients with cancer not cured (15 out of 410, 3.7%) than in patients with cancer cured (1 out of 114, 0.9%) in the LMWH arm (Table 1), suggesting that the risk of major bleeding may be higher in patients with active cancer compared to those with history of cancer, in line with the results of the COMMAND VTE Registry.

Of note, all these studies have examined the rates of recurrent VTE and bleeding in pooled patient populations with different types of cancer. Since significant differences in the clinical course of VTE have been observed according to the site of cancer [31,32], this approach may have failed to capture these differences.

## 4. Conclusions

Few studies separately reported the efficacy and safety of anticoagulant agents in patients with an active cancer compared to those with a history of cancer. Whether these two groups of cancer patients carry the same risk of recurrent VTE and bleeding remains an unanswered issue since no dedicated prospective study has yet addressed this question. Importantly, results from COMMAND-VTE, HOKUSAI VTE Cancer, and AMPLIFY at least suggest that the bleeding risk might be potentially lower in patients with a history of cancer compared to those with an active cancer.

A restrictive definition of “active cancer” has been recently proposed by the SSC on Control of Anticoagulation, and Predictive and Diagnostic Variables in Thrombotic Disease. “Active cancer” is defined as cancer not received potentially curative treatment, or when there is evidence that treatment has not been curative (e.g., recurrent or progressive disease), or when treatment is ongoing [33]. This restrictive definition may reflect more accurately the higher risks of recurrent VTE and bleeding associated with active cancer and should therefore be used in further trials assessing the efficacy and safety of anticoagulants for the prevention of recurrent VTE in cancer patients.

## Figures and Tables

**Table 1:** Risk factors for venous thromboembolism in cancer patients.

Type of Risk Factor	Risk Factor
**Patient-related factors**	Older age Gender (Female) Ethnic origin (higher in African Americans; lower in Asian-Pacific Islanders) Comorbidities (obesity, renal disease, pulmonary disease neutropenia, infection) Prior history of venous thromboembolism Lower performance status Immobilization Heritable prothrombotic mutations (Factor V Leiden, prothrombin gene mutation)
**Cancer-related factors**	Primary tumor site (pancreatic, ovarian, kidney, lung, gastric, brain, and hematologic) Histologic subtype (adenocarcinoma > squamous cell carcinoma) Initial period after diagnosis Locally advanced tumors/distant metastases
**Treatment-related factors**	Recent surgery Hospitalization Central venous catheters Chemotherapy Antiangiogenic agents (bevacizumab, sunitunib, sorafenib) Immunomodulatory drugs (thalidomide, lenalidomide) Hormonal therapy (tamoxifen) Erythropoietin Transfusions (platelets, red blood cells)

**Table 2:** Reported rates of recurrent venous thromboembolism (VTE) and bleeding in patients with an active cancer and in those with an history of cancer.

Study	Sub-group of Patient	Recurrent VTE		Major Bleeding		Major Bleeding and CRNMB		CRNMB	
		Experimental	Control	Experimental	Control	Experimental	Control	Experimental	Control
EINSTEIN-PE and-DVT [27]	Active Cancer at baseline	2% (6/258)	4% (8/204)	2% (5/257)	4% (8/202)	-	-	12% (30/257)	13% (27/202)
	Active Cancer during follow-up	10% (10/96)	12% (12/97)	3% (3/96)	7% (7/96)	-	-	19% (18/96)	23% (22/96)
	History of Cancer	2% (5/233)	2% (5/236)	<1% (1/231)	2% (4/236)	-	-	11% (25/231)	9% (22/236)
AMPLIFY [28]	Active Cancer	3.7% (3/81) *	6.4% (5/78) *	2.3% (2/87)	5.0% (4/80)	12.6% (11/87)	22.5% (18/80)	-	-
	History of Cancer	1.1% (2/179) *	6.3% (11/178) *	0.5% (1/184)	2.8% (5/179)	6.0% (11/184)	15.1% (27/179)	-	-
HOKUSAI [29]	Active Cancer **	4% (4/109)	7 % (7/99)	5% (5/109)	3% (3/99)	18% (20/109)	25% (25/99)	15% (16/109)	23% (23/99)
	Active cancer***	2% (2/85)	9 % (7/77)	5% (4/85)	3% (2/77)	19% (16/85)	26% (20/77)	14% (12/85)	23% (18/77)
	History of Cancer (including patients with active cancer)	4% (14/378)	7 % (28/393)	3 % (10/378)	3% (13/393)	12% (47/378)	19 % (74/393)	10 % (39/378)	16 % (64/393)
COMMAND VTE Registry [30]	Active Cancer	17.7% (78/695)		26.6% (105/695)		-		-	
	History of Cancer	10.2% (18/243)		8.8% (19/243)		-		-	
HOKUSAI VTE Cancer [14]	Cancer non-cured	8.3% (33/397)	11.7% (48/410)	7.3% (29/397) ****	3.7% (15/410) ****	-	-	-	-
	Cancer Cured	6.4% (8/125)	9.6% (11/114)	2.4% (3/125) ****	0.9% (1/114) ****	-	-	-	-
CARAVAGGIO [16]	Active Cancer	5.5% (31/559)	8.1% (46/565)	3.9% (22/559)	3.9% (22/565)	-	-	-	-
	History of Cancer	5.9% (1/17)	0% (0/14)	0% (0/17)	7.1% (1/14)	-	-	-	-

Abbreviations: CRNMB, clinically relevant non-major bleeding; VTE, venous thromboembolism. * VTE + VTE-related death; ** prespecified categorization made by study physician at enrolment; *** post-hoc classification; **** major bleeding in on-treatment safety population.
